# Pedunculopontine-thalamic cholinergic projections in rapid eye movement sleep behaviour disorder

**DOI:** 10.1038/s41531-026-01311-0

**Published:** 2026-03-06

**Authors:** Julia Schumacher, Stefan Teipel, Alexander Storch, Wiebke Hermann

**Affiliations:** 1 Department of Neurology, University of Rostock, Rostock, Germany; 2 Deutsches Zentrum für Neurodegenerative Erkrankungen (DZNE) Rostock-Greifswald, Rostock, Germany; 3 Department of Psychosomatic Medicine, University Medical Center Rostock, Rostock, Germany

**Keywords:** Biomarkers, Diseases, Neurology, Neuroscience

## Abstract

Cholinergic system degeneration is a hallmark of Lewy body disease, including Parkinson’s disease (PD) and dementia with Lewy bodies (DLB), but its involvement in prodromal stages, particularly regarding pedunculopontine-thalamic projections, remains unclear. This study investigated cholinergic pathway integrity in isolated REM sleep behaviour disorder (iRBD) and its relation to phenoconversion risk. We analyzed data from 146 iRBD patients and 102 controls from the Parkinson’s Progression Markers Initiative using T1-weighted MRI for basal forebrain volume and diffusion-weighted imaging for three cholinergic pathways: lateral and medial nucleus basalis of Meynert (NBM)-cortical pathways and pedunculopontine nucleus (PPN)-thalamic pathways. Bayesian mixed models and Cox proportional hazards models assessed group differences, cognitive associations, and phenoconversion risk. Fractional anisotropy along PPN-thalamic pathways was reduced in iRBD, particularly in the left hemisphere. Within iRBD patients, medial NBM pathway integrity correlated with baseline cognitive performance. Reduced PPN-thalamic integrity predicted increased phenoconversion risk (hazard ratio=2.08). No group differences or associations with phenoconversion were found for NBM-cortical pathways or basal forebrain volume. These findings suggest that brainstem-thalamic cholinergic projections may be affected earlier than the basal forebrain system in Lewy body disease, potentially serving as a sensitive marker for phenoconversion risk in iRBD pending validation in longer follow-up studies.

## Introduction

Lewy body disease (LBD) is an umbrella term for a clinico-pathological spectrum encompassing different clinical entities that are characterised by the abnormal accumulation of intraneuronal α-synuclein aggregates in the form of Lewy bodies and Lewy neurites^1^. On one end of this spectrum lies Parkinson’s disease (PD) with α-synuclein pathology mainly within the brainstem and limbic areas and initial motor symptoms^2^. On the other end lies dementia with Lewy bodies (DLB) with more severe neocortical pathology and pronounced cognitive impairment^3^. However, there are no clear boundaries between these diagnostic categories: a majority of PD patients eventually develop dementia^4^, and both PD and DLB are associated with a multiplicity of symptoms ranging from autonomic and motor features to neuropsychiatric and cognitive dysfunction. By the time PD or DLB are diagnosed clinically, the pathological process has potentially been advancing for years and even decades^5^. The prodromal phase of LBD is characterised by many subtle signs and symptoms that are relatively unspecific including hyposmia, neuropsychiatric features, and a range of autonomic symptoms^6^. An exception is clinically isolated rapid eye movement sleep behaviour disorder (iRBD), a parasomnia marked by the loss of muscle atonia during REM sleep with consecutive dream-enactment behaviour confirmed by polysomnography in specialised sleep centres^7^. Longitudinal cohort studies show that most people with iRBD will eventually develop an overt α-synucleinopathy in the form of PD or DLB (and less commonly multiple system atrophy), making iRBD a remarkably specific risk factor for LBD^8–10^. However, RBD is diagnosed in only up to 40–60% of PD patients either as a prodromal feature years or decades before phenoconversion or during the disease course^11^. This led to the pathophysiological concept of a brain-first vs. body-first model of neurodegeneration in PD with iRBD as a marker of the body-first subtype starting in the gut and spreading rostrally until reaching the lower brainstem areas to cause RBD symptoms before PD motor signs occur^12^. Within this prodromal cohort of iRBD, a large inter-individual variation in time to phenoconversion to an overt α-synucleinopathy has been demonstrated, and there is an increasing interest in developing markers of conversion risk for better prognosis and, eventually, stratification of patients for clinical trials with potential neuroprotective drugs.

One of the hallmark features of LBD is a gradual degeneration of the cholinergic system^13,14^. There are two major sources of cholinergic projections in the brain that are affected in LBD. Firstly, the basal forebrain cholinergic nuclei, including the nucleus basalis of Meynert (NBM), provide innervation to the entire cortex as well as the amygdala and hippocampus. Early degeneration of the basal forebrain has been consistently found in DLB and PD^15–17^, as well as in patients with iRBD^18–20^. Furthermore, the NBM-cortical cholinergic projections have been shown to degenerate early in DLB^21^ and PD^22^ and to be associated with cognitive and attentional impairment. Recently, Eisenstein et al. have shown that while the integrity of the NBM-cortical projections in iRBD patients was not different from control levels at baseline, lower levels of integrity were predictive of future phenoconversion^23^. However, while changes of the basal forebrain cholinergic system are an important driver of cognitive impairment in DLB and PD, they are not a specific feature of LBD: in Alzheimer’s disease (AD), similar patterns of basal forebrain atrophy occur^24,25^ and the NBM-cortical pathways are similarly affected^21,26^.

In contrast, the second important source of cholinergic projections, the pedunculopontine nucleus (PPN), might be more specifically affected in LBD. The PPN is located in the brainstem and provides cholinergic input to the thalamus, basal ganglia, lower brainstem areas, and the spinal cord^27,28^. We have previously found the integrity of the PPN-thalamic cholinergic projections to be differentially disrupted in DLB with relative sparing in AD^29^. This is supported by previous PET and post-mortem studies showing cholinergic denervation within the thalamus in DLB and PD, but not AD^30,31^.

The PPN-thalamic projections are largely unexplored in iRBD; however, there is evidence for more severe thalamic cholinergic denervation in PD patients with RBD compared to those without^32^. Furthermore, in those LBD patients who are first diagnosed with iRBD in the prodromal stage (body-first type), the brainstem is thought to be the first brain site of abnormal α-synuclein accumulation^12^. In this study, we therefore aimed to extend the investigation of cholinergic system changes in iRBD to the PPN-thalamic cholinergic system, hypothesising that this is more specifically and severely affected in iRBD than the cholinergic projections from the basal forebrain and might potentially serve as a more sensitive marker of phenoconversion risk.

## Results

### Demographics

The study sample included 146 iRBD patients and 102 control participants. Demographic and clinical information can be found in Table 1. Both groups were similar in age and years of education. The iRBD group had a higher proportion of male participants compared to the control group. UPDRS-III motor scores were slightly increased in the iRBD group at baseline.

**Table 1:** Demographics and clinical information

	iRBD (*N* = 146)	Controls (*N* = 102)	Group comparison
Age at MRI (years), mean (SD)	67.1 (6.2)	66.9 (7.3)	BF_10_ = 0.14^a^
Female:male (%)	26:120 (18:82%)	47:55 (46:54%)	BF_10_ = 1.5*10^4 b^
Years of education	16.3 (3.2)	16.4 (3.2)	BF_10_ = 0.14^a^
UPDRS-III, mean (SD)	3.2 (2.7)	1.3 (1.8)	BF_10_ = 7.2*10^6 a^
MoCA, mean (SD)	27.2 (1.8)	27.7 (1.6)	BF_10_ = 2.0^a^
Follow-up (years), median (range)	2.0 (0–3.9)	-	-
Time since diagnosis (years), median (range)	1.4 (0.17–24.1)	-	-

^a^ Bayesian independent samples T-test.

^b^ Bayesian contingency tables test.

*GDS* Geriatric Depression Scale, *MoCA* Montreal Cognitive Assessment, *RBD* rapid eye movement sleep behaviour disorder, *SD* standard deviation, *UPDRS* Unified Parkinson’s Disease Rating Scale part III (motor score).

During follow-up, 11 of the iRBD patients converted to PD and one patient converted to DLB after a median time of 1.3 years (range: 0.3–3.2 years), while no converters to multiple system atrophy were identified. Due to the low number of DLB converters, we combined PD and DLB converters for the phenoconversion analysis.

### Group comparisons

**Fig. 1:**
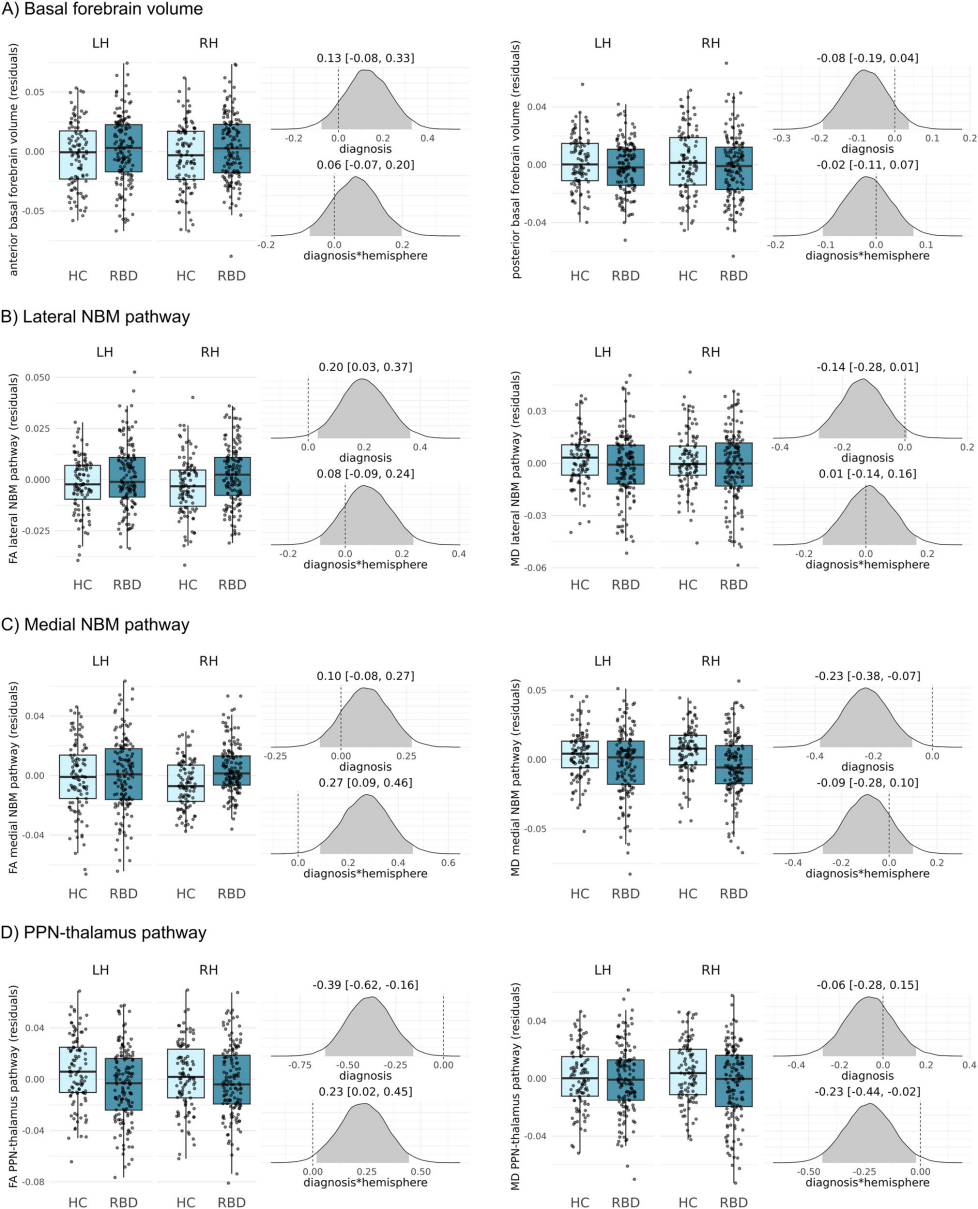
Group comparison of volumetric and DTI metrics. The boxplots show **A** basal forebrain volume, **B** DTI metrics from the lateral NBM pathway, **C** DTI metrics from the medial NBM pathway and **D** DTI metrics from the PPN-thalamus pathway (residuals after regression of age, sex, years of education, and—for DTI metrics—the average DTI metric from the white matter control mask) for left (LH) and right (RH) hemispheres in each diagnostic group. Also shown are the posterior distributions of the standardised parameter estimates for the main effect of diagnosis and the interaction effect of diagnosis*hemisphere from the Bayesian linear mixed-effects models. Shaded areas in the posterior distribution plots indicate the 95% central values of the distribution (credible interval), and above each plot, the median and lower and upper borders of the 95% credible interval are stated. The zero point is indicated by a dashed vertical line. FA fractional anisotropy, HC healthy controls, LH left hemisphere, MD mean diffusivity, NBM nucleus basalis of Meynert, PPN pedunculopontine nucleus, RBD rapid eye movement sleep behaviour disorder, RH right hemisphere.

For anterior and posterior basal forebrain volume, we did not find an effect of diagnosis or a diagnosis*hemisphere interaction as illustrated by the posterior distributions of the parameter estimates largely overlapping with zero (Fig. 1A). Similarly, for the DTI metrics of the lateral NBM pathway, there was no clear evidence for an effect of diagnosis (Fig. 1B). For the medial pathway, MD was reduced in the iRBD cohort, similarly for both hemispheres (95% credible interval of the parameter estimate excluding zero, Fig. 1C). For the PPN-thalamus pathway, we found a reduction in fractional anisotropy in the iRBD cohort as compared to controls (95% credible interval of the parameter estimate excluding zero, Fig. 1D). Furthermore, there was evidence for an interaction between diagnosis and hemisphere, indicating that the effect was stronger for the left PPN-thalamus pathway.

All results remained consistent when using different prior specifications (Supplementary Fig. S1).

### Associations between cholinergic system integrity and cognition

**Fig. 2:**
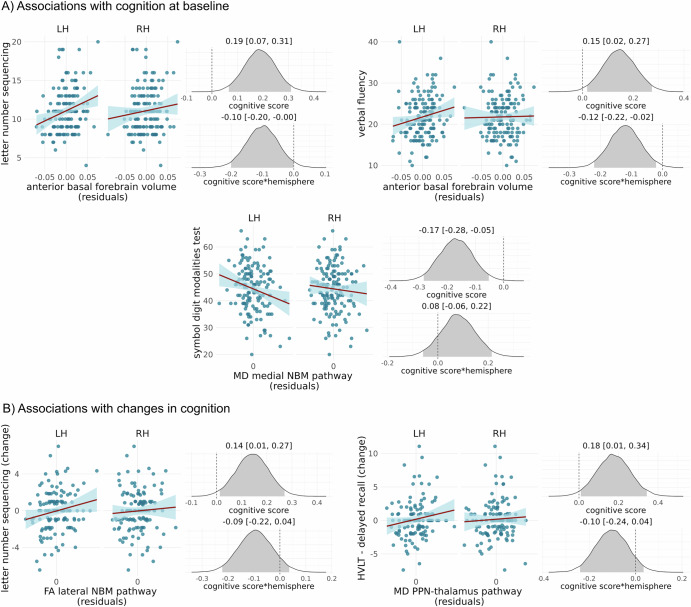
Associations of volumetric and DTI metrics with cognition. Scatter plots show the association between volumetric and DTI metrics (residuals after regression of age, sex, years of education, time between RBD diagnosis and baseline, and—for DTI metrics—the average DTI metric from the white matter control mask) for left (LH) and right (RH) hemispheres with cognitive test scores within the RBD group, **A** at baseline and **B** with changes in cognition over one year. Also shown are the posterior distributions of the standardised parameter estimates for the main effect of cognitive score and the interaction effect of cognitive score*hemisphere from the Bayesian linear mixed-effects models. Shaded areas in the posterior distribution plots indicate the 95% central values of the distribution (credible interval), and above each plot, the median and lower and upper borders of the 95% credible interval are stated. The zero point is indicated by a dashed vertical line. Only associations for which the 95% credible interval for the main effect does not include zero are shown here (full results can be found in Supplementary Figs. S2 and S3). LH left hemisphere, NBM nucleus basalis of Meynert, PPN pedunculopontine nucleus, RH right hemisphere.

At baseline, within the iRBD group, larger anterior basal forebrain volumes were associated with better performance in letter number sequencing and verbal fluency (Fig. 2A). Both analyses showed an interaction effect with hemisphere, indicating that the associations were stronger for the left hemisphere. There was no evidence for an association between posterior basal forebrain volume and cognition.

Lower MD along the medial NBM pathway was associated with better performance on the symbol digit modalities test, similarly for both hemispheres (Fig. 2A).

There was no evidence for an association between the integrity of the PPN-thalamus pathways and cognition in the cross-sectional analysis.

All results remained consistent when using different prior specifications (Supplementary Fig. S2).

### Effect of cholinergic system integrity on changes in cognition

This analysis included 124 iRBD participants who had 1-year follow-up data available. Higher FA along the medial NBM pathway was associated with less decline in letter number sequencing (Fig. 2B). MD along the PPN-thalamus pathway showed an association with change in HVLT delayed recall, with higher MD at baseline being associated with less decline in cognitive performance (Fig. 2B). For all analyses concerning changes in cognition, there was no evidence for an interaction with hemisphere, i.e. effects were similar in both hemispheres.

All results remained consistent when using different prior specifications (Supplementary Fig. S3).

### Association between cholinergic system integrity and phenoconversion

**Fig. 3:**
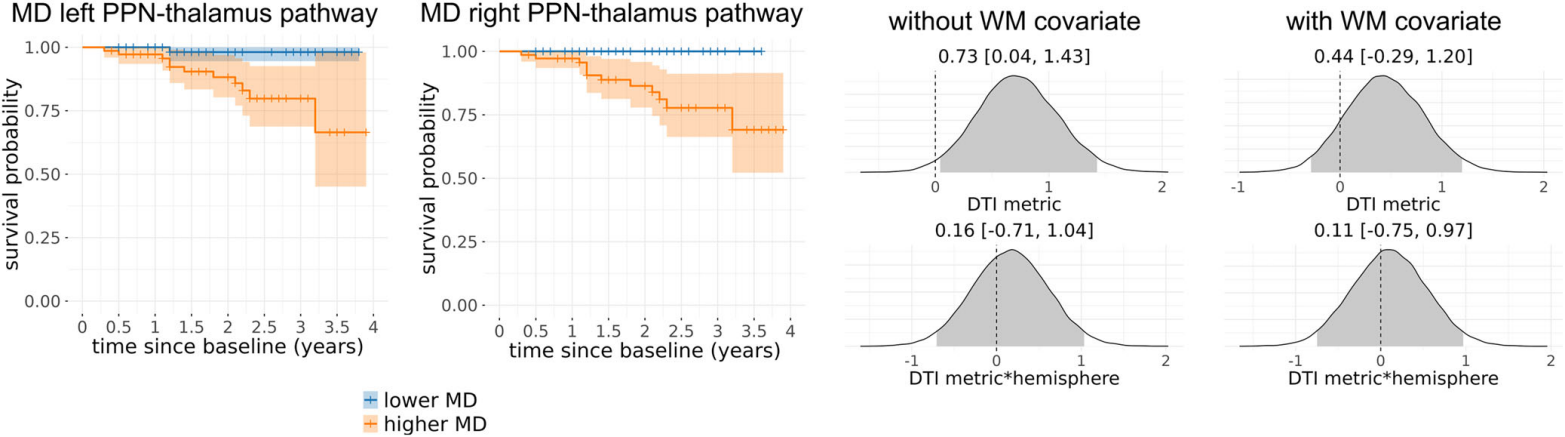
Associations of PPN-thalamus pathway integrity with phenoconversion. Survival curves of conversion to LBD, given baseline MD values along the left and right PPN-thalamus pathways, based on a median split for visualisation purposes. Also shown are posterior distributions of the standardised parameter estimates for the main effect and the interaction with hemisphere from the Bayesian Cox proportional hazards model. Shaded areas in the posterior distribution plots indicate the 95% central values of the distribution (credible interval), and above each plot, the median and lower and upper borders of the 95% credible interval are stated. The zero point is indicated by a dashed vertical line. Results for the other imaging metrics can be found in Supplementary Figs. S4 and S5. MD mean diffusivity, PPN pedunculopontine nucleus.

The analysis of phenoconversion included all iRBD patients who had at least one follow-up visit available (*N* = 144). Basal forebrain volume and the integrity of the NBM pathways did not show any association with phenoconversion in iRBD (Supplementary Figs. S4 and S5). In contrast, elevated MD along the PPN thalamus pathway was associated with an increased risk of conversion to PD/DLB in iRBD patients with a hazard ratio of HR = 2.08 (95% credible interval: 1.04–4.18, Fig. 3), similarly for right and left hemispheres. However, the effect did not persist when controlling for MD from the white matter control mask.

## Discussion

In this study, we investigated the integrity of the two main cholinergic projection systems in iRBD and how changes in these systems relate to cognitive performance and future risk of phenoconversion to an overt α-synucleinopathy. Overall, our findings suggest that in iRBD, degeneration of the PPN-thalamic projections is more pronounced and might be a more sensitive marker of the phenoconversion risk compared to basal forebrain volume and the integrity of NBM-cortical projections.

We found a decrease in FA along the PPN-thalamic projections in the iRBD group compared to controls, i.e. diffusion along these pathways appears more isotropic (non-directional) in iRBD, suggesting a disruption or degeneration of white matter microstructure. Importantly, all analyses of DTI metrics were controlled for general white matter changes, underlining the specificity of these findings to the cholinergic pathways of interest. While the PPN-thalamus cholinergic system has not been extensively studied, particularly in iRBD, previous studies at the LBD stage align well with the present findings. In DLB, studies have found cholinergic denervation within the thalamus^31,33^ and a reduction in the integrity of the PPN-thalamus pathway^29^. Notably, in contrast to the basal forebrain cholinergic system, which is similarly affected in DLB and AD, this loss of cholinergic input to the thalamus appears specific to DLB with relative sparing in AD^29,31^. In PD, cholinergic denervation within the thalamus has been shown to be more severe in patients with RBD compared to those without RBD^32^. Taken together with the present findings, this indicates a gradual degeneration of the PPN cholinergic projection system in LBD, beginning particularly early in those patients who initially present as iRBD. This fits with the brain-first vs. body-first model of α-synuclein pathology accumulation in which the symptom of RBD is postulated to be an indicator of the body-first subtype with α-synuclein pathology starting in the peripheral nervous system, entering the brain via the brainstem and then invading the rest of the brain in an ascending manner^12^.

In addition to the effect of diagnosis, we observed evidence for a lateralisation effect, with more pronounced changes in the left compared to the right PPN-thalamus pathway. Motor symptoms in PD typically start unilaterally with the dominant side of the body often being affected first, mapping to changes in the contralateral brain hemisphere^34^. A corresponding hemispheric asymmetry has been described for the dopaminergic system already at the iRBD stage, where subclinical nigrostriatal dysfunction as well as longitudinal rate of decline have been shown to be more pronounced in the left hemisphere in right-handed patients^35,36^. Most patients in our study cohort were right-handed individuals (*N* = 127). The observed difference between left and right PPN-thalamus pathway integrity might therefore indicate a similar hemispheric asymmetry for changes in the brainstem cholinergic system in early LBD. In a post-hoc analysis, we tested the association between motor asymmetry on the UPDRS and asymmetry of the PPN-thalamus pathway, which did not show an effect (Supplementary Fig. S6). Assessment of motor asymmetry, however, is not very reliable in this cohort as all iRBD patients had very low UPDRS scores at baseline. Future studies in RBD patients with more advanced motor symptoms or in a Parkinson’s disease cohort are therefore required to test the association between asymmetry of cholinergic system degeneration and clinical asymmetry.

While PPN-thalamic projection integrity was not associated with cognitive performance cross-sectionally, we found an unexpected association between lower MD of the PPN-thalamus pathway and worse decline on a memory test. This result could be confounded by the relatively large number of iRBD patients who showed a better performance after 1 year compared to baseline. Furthermore, the longitudinal associations are based on relatively short follow-up times and should be interpreted with caution until confirmed in cohorts with longer follow-up data.

A reduction in the integrity of PPN-thalamic projections was related to an increased risk of future phenoconversion in iRBD, with a one standard deviation increase in mean diffusivity along this pathway approximately doubling the hazard of conversion to LBD. This indicates that microstructural integrity of the PPN-thalamus pathway is not only an early marker of neurodegeneration within the cholinergic system in iRBD, but could also serve as a marker to identify individuals at high risk of converting to clinically defined PD or DLB. Such a marker might be relevant for prognosis in individual patients as well as for stratification of patients for clinical trials when disease-modifying or neuroprotective treatments become available. However, it should be noted that this finding did not persist when controlling for general white matter changes, which could be due to low statistical power due to a small number of conversion events, but might also question the specificity of this finding for the PPN-thalamus tract. The PPMI iRBD cohort is still in the early stages of longitudinal data collection, leading to short follow-up times and a relatively low number of phenoconverters. The potential prognostic value of cholinergic imaging metrics in iRBD, therefore, warrants confirmation in longer-term longitudinal cohorts in the future.

We did not find evidence for a reduction of basal forebrain volume or the integrity of the two NBM-cortical pathways in iRBD compared to similarly aged controls. In contrast, MD in the medial NBM tract was even slightly lower in the iRBD group. The finding of preserved integrity of the NBM pathways in iRBD is consistent with the only previous study on this topic^23^. With respect to basal forebrain volume, results from previous studies are conflicting and appear to be influenced by the disease duration of the respective iRBD cohort. Studies reporting a reduction in basal forebrain volume in iRBD tend to include participants with longer average disease duration (Eisenstein et al*.*: 2.3 years^18^; Kim et al.: 5 years^19^; Yang and Li: 2.7 years^20^) compared to those studies that did not find group differences (Woo et al.: 1.5 years^37^; Churchill et al.: 1.9 years^38^). This suggests that changes in the basal forebrain cholinergic system progress during the course of iRBD until they reach a level that is detectable with volumetric analyses, which might explain the lack of group differences in our study group with relatively short disease duration. However, longitudinal imaging data would be required to confirm this conjecture. Furthermore, it should be noted that the assessment of disease duration in iRBD patients is a challenge in itself. Since RBD symptoms might be subtle with only minor movements (“jerks”) during REM sleep or vocalisations and are often only observed by bedpartners, the clinical relevance might be overlooked by patients, caretakers and medical professionals, particularly in patients without bedpartners or spouses. Thus, symptom as well as diagnosis duration is variable and often does not reflect the actual symptom duration accurately.

Despite showing no changes compared to controls, the basal forebrain cholinergic system, i.e. basal forebrain volume as well as the integrity of the medial NBM pathway, was clearly associated with cognitive performance in the iRBD group, including tests of processing speed, executive, and language function. While changes in the NBM-cortical connections are not severe enough at the group level in iRBD patients to show clear differences to controls, individual patients do show degeneration in this system, which relates to subtle impairments in cognitive processes that are driven by the cholinergic system, consistent with previous studies^18,21,23,29,38^.

Since recruitment of iRBD patients in PPMI only started in 2021, the follow-up times are relatively short, and the number of phenoconverters is low, which might have prevented us from seeing an association between the integrity of the basal forebrain system and phenoconversion as observed in other cohorts^18,23,38^. It is very conceivable that with longer follow-up durations and a larger number of phenoconverters, this relationship will also become apparent in the PPMI cohort. Nevertheless, the fact that the integrity of the PPN-thalamic projections is already altered and predictive of phenoconversion in this sample with limited longitudinal data indicates the potential higher sensitivity of this imaging marker to very early changes in LBD patients. In a larger cohort with more DLB converters, it will be interesting to investigate potential differences in the two cholinergic projection systems in individuals who progress to DLB compared to those who progress to PD.

In contrast to previous studies, we excluded patients from the iRBD group with marked motor or cognitive impairment to ensure a more *clinically isolated* RBD sample. For the MoCA score, we used an established threshold, resulting in a distribution of MoCA scores in the iRBD sample which was similar to the control group^39,40^. For UPDRS III scores, there is no consensus on an appropriate cut-off in isolated RBD patients, so we adopted a pragmatic threshold balancing the risk of excluding too many patients against including those with advanced motor impairment. The resulting distribution of UPDRS III scores in the iRBD group—while slightly higher compared to our control group—is comparable to other studies in healthy older controls^41^ and in large iRBD samples from recent studies^42,43^.

PPMI data were acquired at different study sites, on different scanner models and with different acquisition protocols. To mitigate any site/scanner effects, we applied ComBat, a harmonisation technique that has been shown to effectively remove unwanted technical between-scanner variation while preserving the between-subject biological variability of interest^44^ and has been widely applied in the field of MRI^45^. An assessment of the PPN itself is difficult with in vivo neuroimaging data due to its small size and location in the brainstem. Nevertheless, some previous studies have attempted an investigation of PPN integrity using DTI^46,47^. However, a lot of clinical DWI data, including those from PPMI, have a resolution that is not high enough to assess such a small structure. We therefore focused our analysis on the PPN-thalamic pathways instead of the PPN itself. In addition to its population of cholinergic neurons, the PPN also contains glutamatergic and GABAergic neurons^48^. We can therefore not rule out the possibility that the PPN mask that we used as the seed region for the tractography analysis contained some non-cholinergic neurons. However, the PPN-thalamic projections have been shown to be predominantly cholinergic^49^. The analysed NBM-cortical tracts were similar to previously identified tracts from an immunohistochemistry study in which the cholinergic nature of these tracts was confirmed^50^.

This study offers new insights into early alterations within the complex structure of the cholinergic system in LBD, highlighting that the cholinergic projections from the brainstem to the thalamus may be affected even earlier than the basal forebrain cholinergic system and—if confirmed in cohorts with longer follow-up times—might serve as a sensitive marker for increased risk of phenoconversion in iRBD.

## Methods

### Participants

Data used in the preparation of this article were openly available from the Parkinson’s Progression Markers Initiative (PPMI) database (www.ppmi-info.org/access-data-specimens/download-data). For up-to-date information on the study, visit www.ppmi-info.org. We included all participants with a polysomnography-confirmed diagnosis of RBD at baseline, no diagnosis of PD or dementia at baseline and good quality T1-weighted MRI and diffusion weighted imaging data. Despite having no clinical diagnosis of PD, some iRBD patients had relatively high scores on Unified Parkinson’s Disease Rating Scale (UPDRS) III motor examination, questioning whether they are still *clinically isolated* RBD cases. A pragmatic cut-off score for the UPDRS-III was therefore chosen, excluding all patients with scores above 10 from the iRBD cohort. Furthermore, despite having no clinical dementia diagnosis, some iRBD patients showed very low scores on cognitive testing. We therefore excluded participants with a Montreal Cognitive Assessment (MoCA) score below 22^39,40^. As a comparison group, we included healthy controls from PPMI who were similar in age, excluding controls who were <50 years of age, as this was the minimum age in the iRBD group.

The PPMI study was conducted in accordance with the Good Clinical Practice guidelines and was approved by the local ethics committee at each participating centre. These included the University of Michigan, the National and Kapodistrian University of Athens, Emory University School of Medicine, University of Colorado Denver, Johns Hopkins University, Hospital Clinic de Barcelona, University of Alabama at Birmingham, Parkinson’s Disease and Movement Disorders Center of Boca Raton, Massachusetts General Hospital, Boston University Medical Center, Northwestern University, University of Cincinnati, The Cleveland Clinic, Hospital Universitario Donostia, University of Florida, Baylor College of Medicine, Medical University Innsbruck, University of Kansas Medical Center, Paracelsus-Elena Klinik Kassel, University of California San Diego, Lagos College of Medicine, Cleveland Clinic Lou Ruvo Center for Brain Health, Imperial College London, Queen Mary East London, Keck School of Medicine of USC, University of Luebeck, University of Luxembourg, Philipps-University of Marburg, Montreal Neurological Institute-Hospital, Institute for Neurodegenerative Disorders/XingImaging, Mount Sinai Beth Israel, NYU Langone Health, Clinical Ageing Research Unit, Newcastle University, Radboud University, The Ottawa Hospital, John Radcliffe Hospital Oxford and Oxford University, University of Pennsylvania, Barrow Neurological Institute, University of Pittsburgh, Oregon Health and Science University, University of Rochester, University of Salerno, University of California San Francisco, Mayo Clinic of Arizona, VA Puget Sound Health Care System, Banner Sun Health Research Institute, University of South Florida, Tel Aviv Sourasky Medical Center, Toronto Western Hospital, University of Tuebingen. Written informed consent was obtained from each participant prior to inclusion in the study.

### MRI acquisition and preprocessing

T1-weighted 3D volumetric MR images in PPMI were acquired on different GE, Philips, and Siemens scanners with an MPRAGE or IR-FSPGR sequence and a slice thickness of 2 mm or less. We only included data from 3T scanners in our analysis. T1-weighted MR images were segmented into grey matter, white matter, and cerebrospinal fluid, and spatially normalised to MNI space using the CAT12 toolbox in SPM12 (http://www.fil.ion.ucl.ac.uk/spm/). Voxel values of spatially normalised grey matter maps were modulated by the Jacobian determinant of the deformation parameters in order to preserve the volume present in native space.

### Analysis of basal forebrain volume

**Fig. 4:**
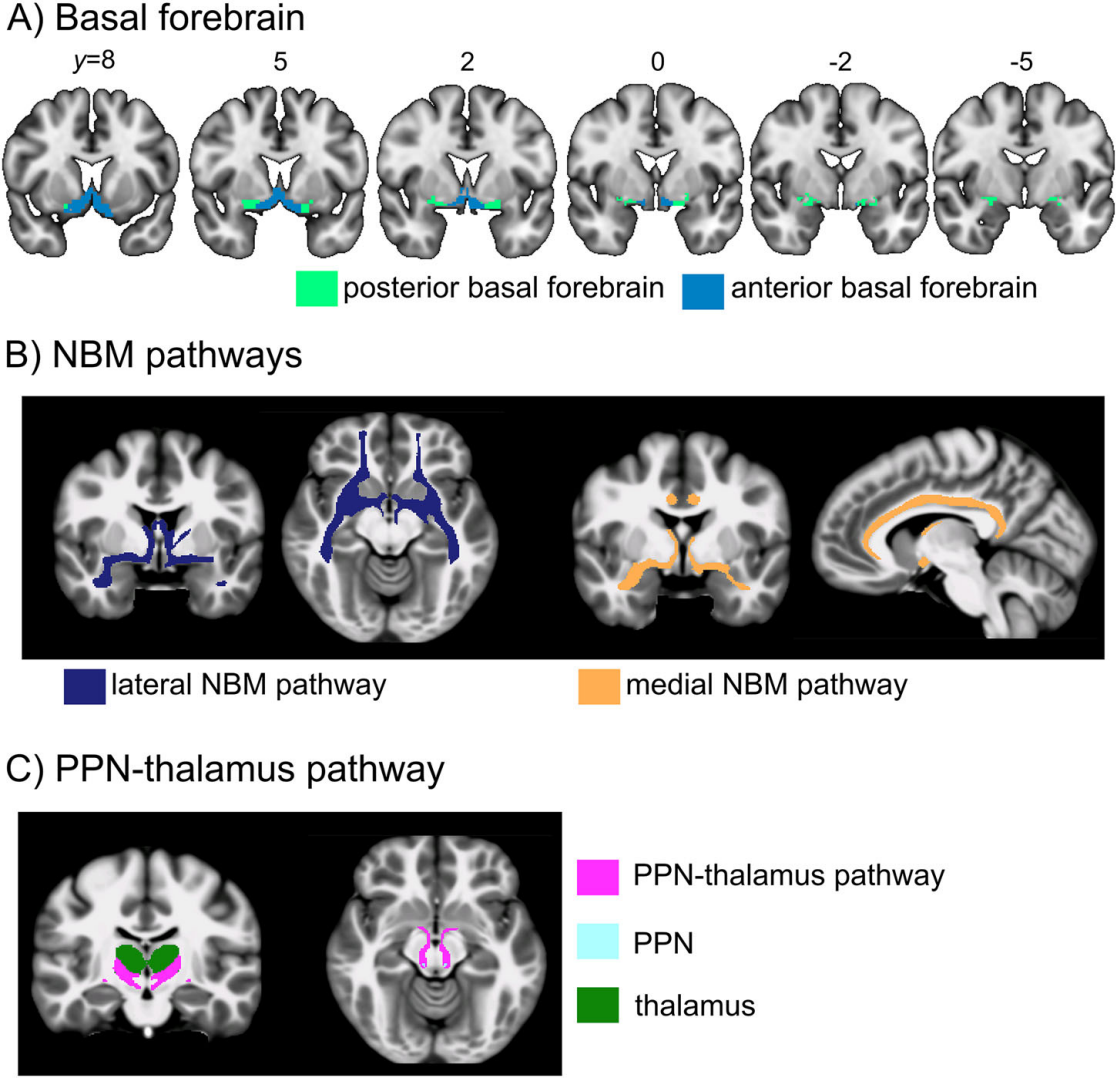
Brain regions and white matter tracts of interest. **A** Basal forebrain mask from Fritz et al. **B** Templates for the lateral and medial NBM pathways, and **C** template for the PPN-thalamus pathway, estimated on high-quality DTI data from the Human Connectome Project. NBM nucleus basalis of Meynert, PPN pedunculopontine nucleus.

The volume of the basal forebrain was estimated from the normalised grey matter images by summing up the modulated grey matter values within a consensus ROI combining information from existing cytoarchitectonic maps of basal forebrain cholinergic nuclei in MNI space, which have been derived from combined histology and MRI of post-mortem brains^51–54^. We estimated the volume of two basal forebrain sub-regions that were identified based on their differential cortical connectivity profile in resting state fMRI data^52^. In this functionally defined subdivision, the posterior basal forebrain mainly corresponds to the cytoarchitectonic sub-region of the nucleus basalis of Meynert (NBM) while the anterior basal forebrain covers the medial septum and diagonal band of Broca (see Fig. 4A).

### DWI acquisition and preprocessing

DWI data for the PPMI participants included in this analysis were acquired on 3T scanners with 30–70 gradient directions with *b* = 1000 s/mm^2^ and one or more *b* = 0 s/mm^2^ images. Some participants had undergone multi-shell DWI acquisitions with multiple non-zero *b*-values; however, for the present analysis, only the *b* = 1000 data were selected to ensure comparability across participants. DWI data were preprocessed using functions from the FMRIB software library (FSL) version 6.0.3 and from MRtrix3 (https://www.mrtrix.org/). First, data were denoised, and Gibbs ringing artefacts were removed using MRtrix’ *dwidenoise* and *mrdegibbs*. Since not all acquisitions included a second B0 image with reversed phase encoding direction, a synthetic B0 image was generated using the SynB0-DISCO algorithm for every participant^55,56^. This “undistorted” image was subsequently used to correct for susceptibility-induced distortions using FSL’s *topup* in combination with FSL’s *eddy* to correct for eddy currents. Finally, images were bias-corrected with MRtrix’ *dwibiascorrect*. Brain extraction was performed on the B0 images using *SynthStrip* from Freesurfer^57^. Transformations from subject DWI space to MNI standard space were estimated with Advanced Normalisation Tools’ (ANTs) non-linear SyN algorithm. Furthermore, co-registration parameters between individual DWI and T1 space were calculated with ANTs using affine registration.

### Regions of interest for tractography analysis

The NBM seed region was based on the Ch4 region of a cytoarchitectonic map of the cholinergic basal forebrain in MNI space that had been derived from combined histology and MRI of a post-mortem brain^51^. From the NBM, we examined a medial pathway travelling through the cingulum toward cingulate, retrosplenial and subcallosal cortex, and a lateral pathway travelling through the external capsule and uncinate fasciculus to innervate the insula, frontal, parietal and temporal cortex^21,50,58^. The tractography was guided by waypoint masks in the cingulum for the medial and in the external capsule for the lateral pathway, which were both obtained from the Johns Hopkins University white matter atlas in FSL. To avoid contamination of estimated tracts from non-cholinergic pathways, we used masks for the anterior commissure (obtained from FSLs’ XTRACT tool) and the brainstem (estimated using FSL’s FIRST segmentation routine) as exclusion masks. Since most neurons from the NBM project to the ipsilateral hemisphere^59^, a mask for the contralateral hemisphere was used to prevent tracts from crossing over. Finally, the waypoint mask of the other pathway was used as an additional exclusion mask (i.e. excluding the external capsule from the medial and the cingulum from the lateral pathway).

For the PPN-thalamus pathway, we performed seed-to-target tractography using the PPN mask from the Brainstem Navigator Atlas^60^ as the seed region and the thalamus mask from the AAL3 atlas^61^ as the target region. Exclusion ROIs for the PPN-thalamus tractography included inferior, middle, and superior cerebellar peduncle (from the Johns Hopkins University white matter atlas) and caudate, putamen, and pallidum (from the Harvard-Oxford Atlas) to avoid pathways from the PPN toward lower brainstem nuclei, down to the spinal cord, and to the basal ganglia^29^. The contralateral hemisphere was used as an additional exclusion mask since most cholinergic projections from the PPN to the thalamus run ipsilaterally^49^.

### Creation of cholinergic pathway templates

To estimate templates for the cholinergic pathways, we used data from the Human Connectome Project (HCP) Young Adult cohort, including 100 unrelated participants aged 22–35^62^. High-quality DWI data in the HCP were acquired on a single Siemens Skyra Connectom scanner using a High Angular Resolution Diffusion Imaging sequence with a multiband factor of 3, isotropic voxel size of 1.25 mm and 270 diffusion directions distributed over three shells with *b*-values of 1000, 2000 and 3000 s/mm^2^ with 18 B0 scans. Each scan was repeated with a reversed phase encoding direction. We downloaded preprocessed DWI data that were already susceptibility and eddy-current corrected according to the HCP protocol^63^.

The response functions were estimated using MRtrix’ *dwi2response* with the *dhollander* option, followed by estimation of the fibre orientation distribution (FOD) using *dwi2fod* with the *msmt_csd* algorithm. FODs were intensity normalised. A group template was generated using the average white matter FOD images from all participants and normalised to MNI standard space using ANTs non-linear Syn algorithm.

All seed, target, and exclusion region maps were transformed to native space using the previously estimated parameters using ANTs. The respective tracts were then estimated for every participant in native space separately for left and right hemispheres using MRtrix’ *tckgen*, transformed to the group template space, and thresholded (retaining voxels that were part of the tract in at least 90% of participants) and binarized. Finally, group tracts were transformed to MNI space with ANTs. An illustration of the tract templates for the lateral and medial NBM as well as the PPN-thalamus pathways can be found in Fig. 4B, C.

### Analysis of cholinergic pathway integrity

A DTI model was fit to the final preprocessed DWI data from PPMI using FSL’s dtifit algorithm. The cholinergic tract templates from HCP data were brought from MNI space into each participant’s native space using the previously estimated ANTs transformations. Parts of the tracts of interest run close to the ventricles, which might lead to spill-in of CSF signal, e.g. in case of ventricular dilation. To address this, FSL FAST was run on each subject’s T1-weighted image to create a subject-specific mask of CSF voxels, which was then transformed to DWI space using the transformation parameters from the coregistration between T1-weighted and DWI data. CSF voxels were then excluded from all tracts, and average DTI metrics (mean diffusivity, MD, and fractional anisotropy, FA) were extracted.

To control for general white matter changes, a white matter control mask was created by subtracting the respective tract from a whole-brain white matter mask obtained from FAST^21^. Mean MD and FA were calculated within the white matter control masks and used as covariates in subsequent analyses.

### Imaging data harmonisation

To account for the fact that PPMI data were acquired at different sites on different scanners, we performed ComBat harmonization on all imaging metrics (volumetric and DTI measures) using the PPMI site as the batch ID^44,64^. All subsequent analyses were then conducted using the ComBat-harmonised values.

### Neuropsychological assessment

Participants underwent a detailed neuropsychological assessment covering different cognitive domains: Montreal Cognitive Assessment (MoCA, global cognition), Benton judgement of line orientation (visuospatial function), letter number sequencing (executive function/working memory), symbol digit modalities test (processing speed/attention), animal verbal fluency test (executive function/language), and Hopkins Verbal Learning Test-Revised (HVLT, episodic memory). These assessments were conducted at baseline and during annual follow-up visits.

### Phenoconversion

All iRBD participants’ follow-up clinical data were evaluated for evidence of phenoconversion, i.e. a clinical diagnosis of Parkinson’s disease (PD) or dementia with Lewy bodies (DLB) at a follow-up visit. Thus, time to phenoconversion was determined as time from baseline until the first documentation of a PD or DLB diagnosis. No iRBD patient converted to multiple system atrophy in this cohort.

### Statistical analysis

All statistical analyses were performed in a Bayesian framework, which is an approach to parameter estimation based on Bayes’ theorem, where prior knowledge about parameters of a statistical model is updated with the information obtained from observed data. A Bayesian analysis, therefore, consists of deriving a posterior distribution of the parameters of interest from the combination of a prior distribution and the model likelihood estimated from the data^65^. This framework is increasingly being adopted across many scientific fields, including medical research, due to its advantages over the classical frequentist approach, which include the ability to directly compare several hypotheses and the possibility of a quantitative interpretation of evidence beyond the classical dichotomisation into significant and non-significant^66^. A key strength of Bayesian hypothesis testing as opposed to the frequentist approach is that it provides the possibility to directly quantify support in favour of the null hypothesis, not only against it^67^.

For Bayesian statistical analysis, we used Jeffreys’s Amazing Statistics Program (JASP, version 0.17.1) and the brms package (version 2.20.1)^68^ in R (https://www.r-project.org/). For group comparison of demographic variables, we report Bayes Factor (BF_10_)^69^ to quantify evidence in favour of the alternative over the null hypothesis^67^. The Bayes Factor is interpreted as the relative likelihood of the data under the models of interest, i.e. BF_10_ quantifies the likelihood of the data given H1 compared to the likelihood of the data given H0. For all other models, we show posterior distributions of the standardised parameter estimates with the median and 95% credible interval.

Group comparisons of left- and right-hemispheric volumetric and DTI measures between iRBD patients and controls were conducted using a Bayesian multivariate linear mixed-effects model including all eight neuroimaging measures and explicitly modelling the residual correlations among the multivariate outcomes. For each outcome, the model included fixed effects for diagnosis (iRBD vs controls), hemisphere, and the interaction between diagnosis and hemisphere, a varying intercept for each participant, and covariates for age, sex and years of education. For the group comparison of DTI metrics, an additional covariate was included for the mean DTI metric from the white matter control mask.

To assess cross-sectional associations between imaging measures and cognition, a Bayesian multivariate linear mixed-effects model for each cognitive test was conducted in the iRBD group separately with fixed effects for the cognitive test score, hemisphere, and the interaction between test score and hemisphere, a varying intercept for each participant, and covariates for age, sex, years of education, and time between RBD diagnosis and baseline visit. The mean DTI metric from the white matter control mask was included as an additional covariate for analyses of DTI metrics.

Longitudinal changes in cognition were examined in a similar way to the cross-sectional analysis, but using changes in cognitive test scores from baseline to the 12-month follow-up visit, divided by the time between the two visits in years. An additional covariate was included in these analyses for the baseline cognitive score.

To assess the robustness of our findings, we fitted the multivariate Bayesian linear mixed models using three alternative prior specifications (Supplementary Table S1). The baseline specification used weakly informative normal priors (mean = 0, standard deviation = 1) for fixed effects and intercepts, and exponential(1) priors for standard deviation parameters and residual variance. In a second specification, we replaced the normal priors on fixed effects with heavier-tailed Student-t priors to reduce sensitivity to potential outliers and test the robustness of small effects. In a third specification, we applied stronger regularisation to variance components to induce moderate shrinkage of correlation estimates.

To examine associations between imaging metrics and time to conversion to PD/DLB, we used Bayesian Cox proportional hazards models. The survival time was defined as the time interval between baseline and PD/DLB conversion, with right-censoring for participants who had not converted at their last follow-up visit. We included fixed effects for the volumetric/DTI metric, hemisphere, and the interaction between imaging metric and hemisphere, covariates for age, sex, years of education, time from RBD diagnosis to baseline, and—for the DTI metrics—the average DTI metric from the white matter control mask, and a clustering term at the participant level. Weakly informative normal priors with mean 0 and standard deviation 1 were used for all regression coefficients to provide mild regularisation and stabilise estimation without strongly influencing the posterior.

## Supplementary information

SupplementaryMaterial

## Data Availability

Data used in the preparation of this article were obtained on July 8th 2025, from the Parkinson’s Progression Markers Initiative (PPMI) database (www.ppmi-info.org/access-dataspecimens/ download-data), RRID:SCR_006431. For up-to-date information on the study, visit www.ppmi-info.org.
