# Supplementary material for: Pedunculopontine-thalamic cholinergic projections in rapid eye movement sleep behaviour disorder
Source: NPJ Parkinsons Dis. 2026 Mar 6;12:67. doi: 10.1038/s41531-026-01311-0 (PMC12992781; doi:10.1038/s41531-026-01311-0)
Supplement: Supplementary file 1 — SupplementaryMaterial [file 41531_2026_1311_MOESM1_ESM.pdf]

Supplementary Material

Pedunculopontine-thalamic cholinergic projections in rapid eye movement sleep behaviour disorder

Julia Schumacher[1,2], Stefan Teipel[1,3], Alexander Storch[1,2], Wiebke Hermann[1]

[1] Department of Neurology, University of Rostock, 18147 Rostock, Germany

[2] Deutsches Zentrum für Neurodegenerative Erkrankungen (DZNE) Rostock-Greifswald, 18147 Rostock, Germany

[3] Department of Psychosomatic Medicine, University Medical Center Rostock, 18147 Rostock, Germany

Correspondence to:

Julia Schumacher

Klinik für Neurologie, Universitätsmedizin Rostock, Gehlsheimer Str. 20, 18147 Rostock, Germany; Email: julia.schumacher@med.uni-rostock.de

Supplementary Table S1: Prior specifications that were used for the Bayesian multivariate linear mixed models.

Prior	Specification
Baseline	Fixed effects: Normal(0,1)
	Intercept: Normal(0,1)
	Standard deviations: Exponential(1)
Heavy tail	Fixed effects: Student-t(3,0,1)
	Intercept: Normal(0,1)
	Standard deviations: Exponential(1)
Correlation shrinkage	Fixed effects: Normal(0,1)
	Intercept: Normal(0,1)
	Standard deviations: Exponential(1)
	Residual correlations: LKJ(4)

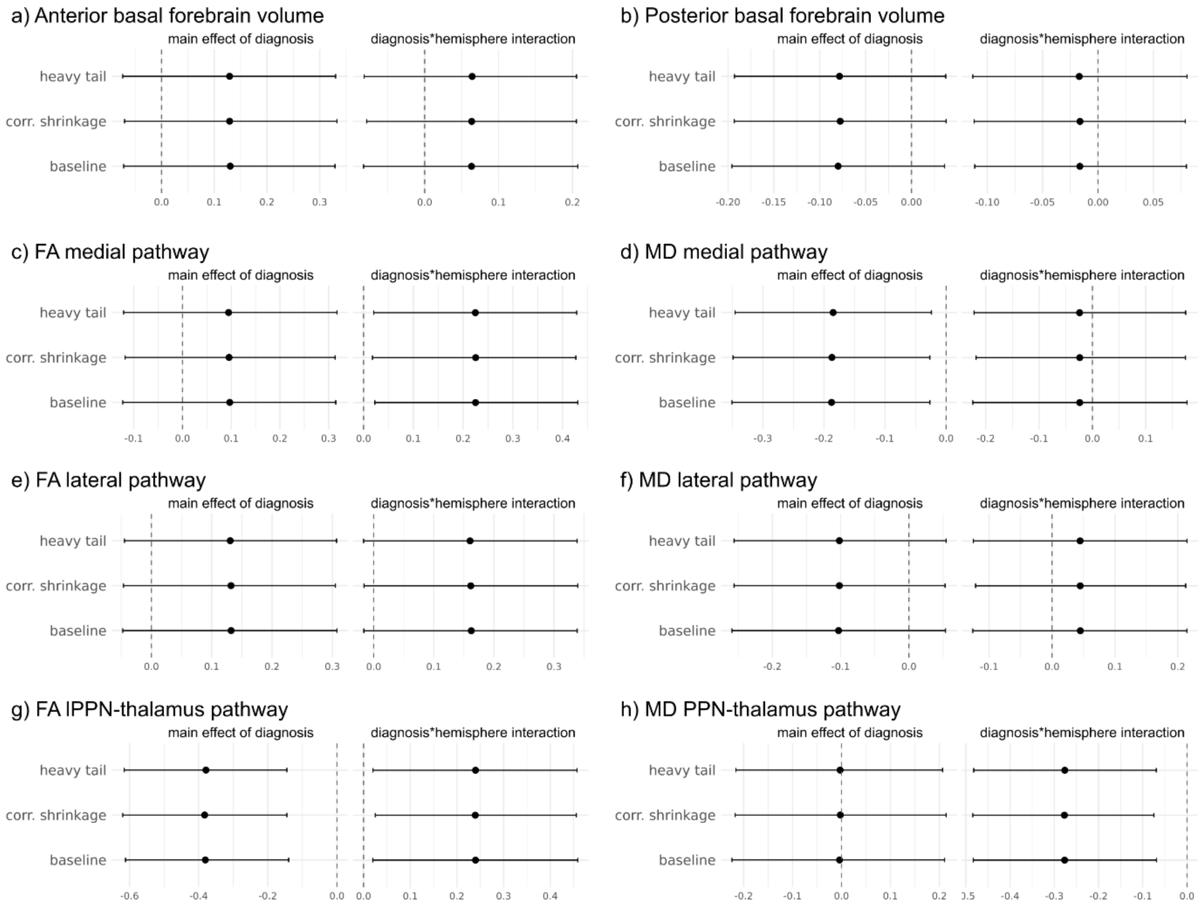

Supplementary Figure S1: Group comparison of volumetric and DTI metrics with different prior specifications. Median and 95% credible intervals of the posterior distributions of the standardised parameter estimates for the main effect of diagnosis and the interaction effect of diagnosis*hemisphere from Bayesian linear mixed models. The zero point is indicated by a vertical dashed line.

FA, fractional anisotropy; MD, mean diffusivity; PPN, pedunculopontine nucleus

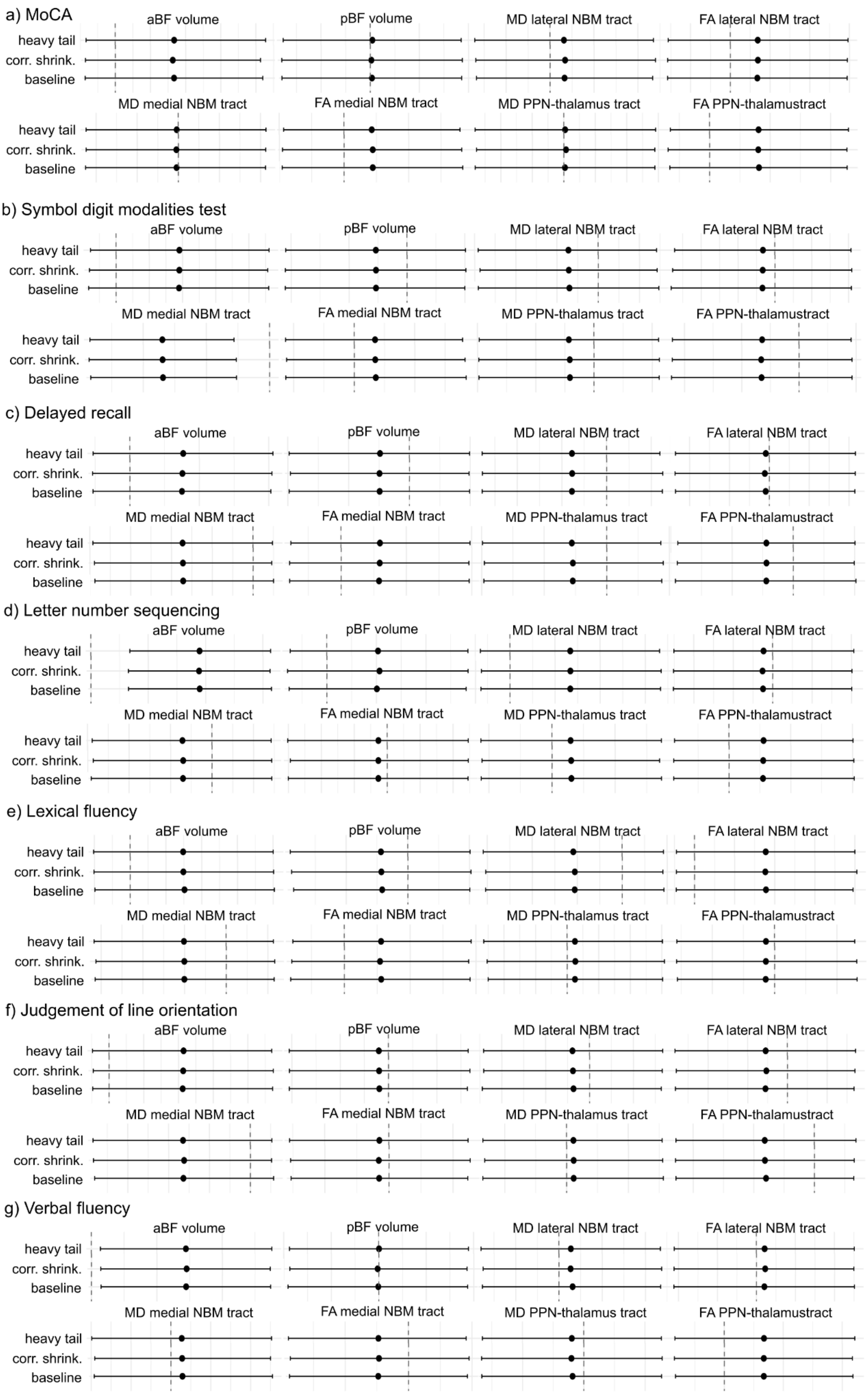

Supplementary Figure S2: Associations of volumetric and DTI metrics with cognition at baseline. Median and 95% credible intervals of the posterior distributions of the standardised parameter estimates for the main effect of cognitive score from Bayesian linear mixed models. The zero point is indicated by a vertical dashed

line.
aBF, anterior basal forebrain; FA, fractional anisotropy; HVLT, Hopkins Verbal Learning Test-Revised; MD, mean diffusivity; MoCA, Montreal Cognitive Assessment; NBM, nucleus basalis of Meynert; pBF, posterior basal forebrain; PPN, pedunculopontine nucleus

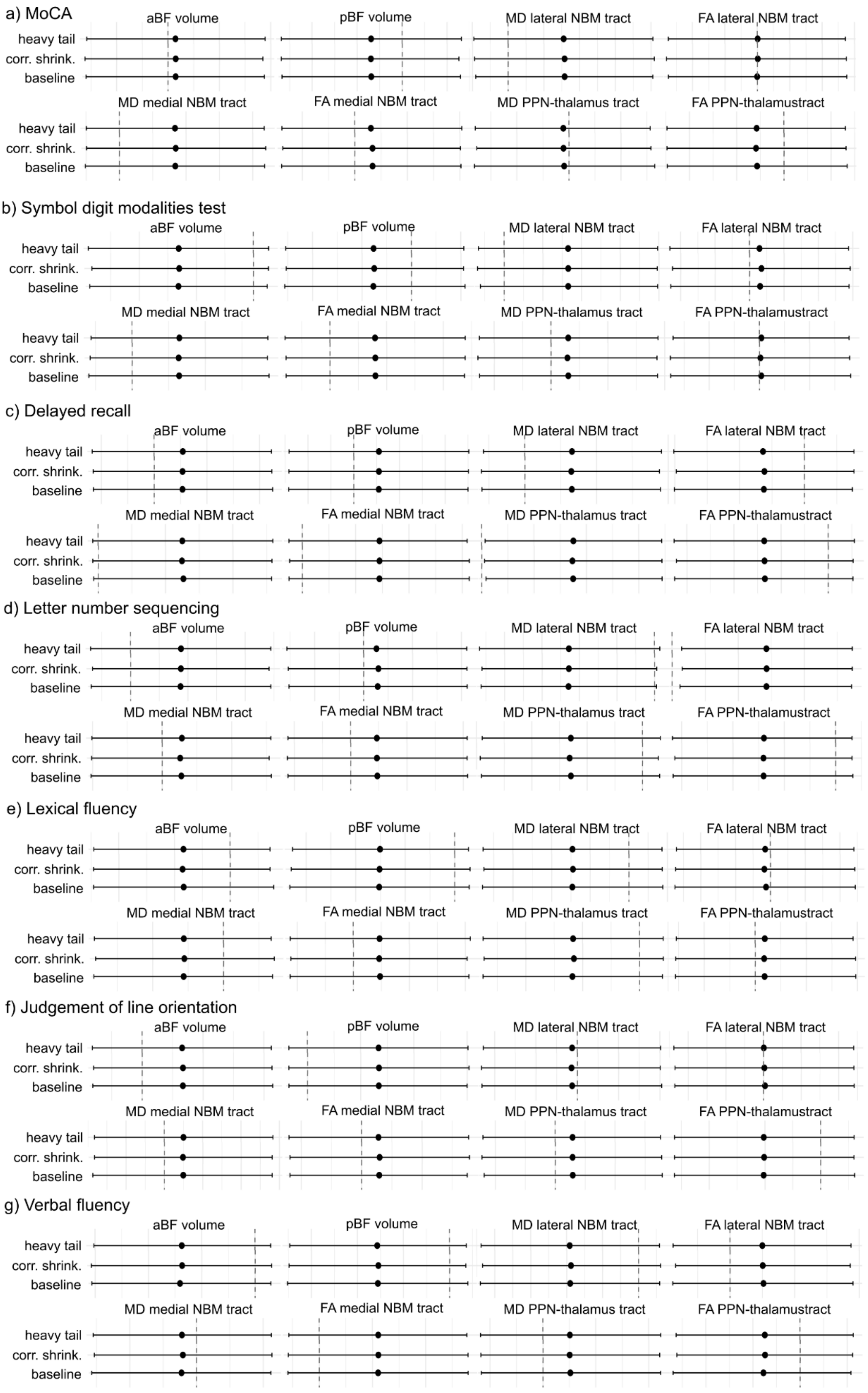

Supplementary Figure S3: Associations of volumetric and DTI metrics with changes in cognition over one year. Median and 95% credible intervals of the posterior distributions of the standardised parameter estimates

for the main effect of cognitive score from Bayesian linear mixed models. The zero point is indicated by a vertical dashed line.

aBF, anterior basal forebrain; FA, fractional anisotropy; HVLT, Hopkins Verbal Learning Test-Revised; MD, mean diffusivity; MoCA, Montreal Cognitive Assessment; NBM, nucleus basalis of Meynert; pBF, posterior basal forebrain; PPN, pedunculopontine nucleus

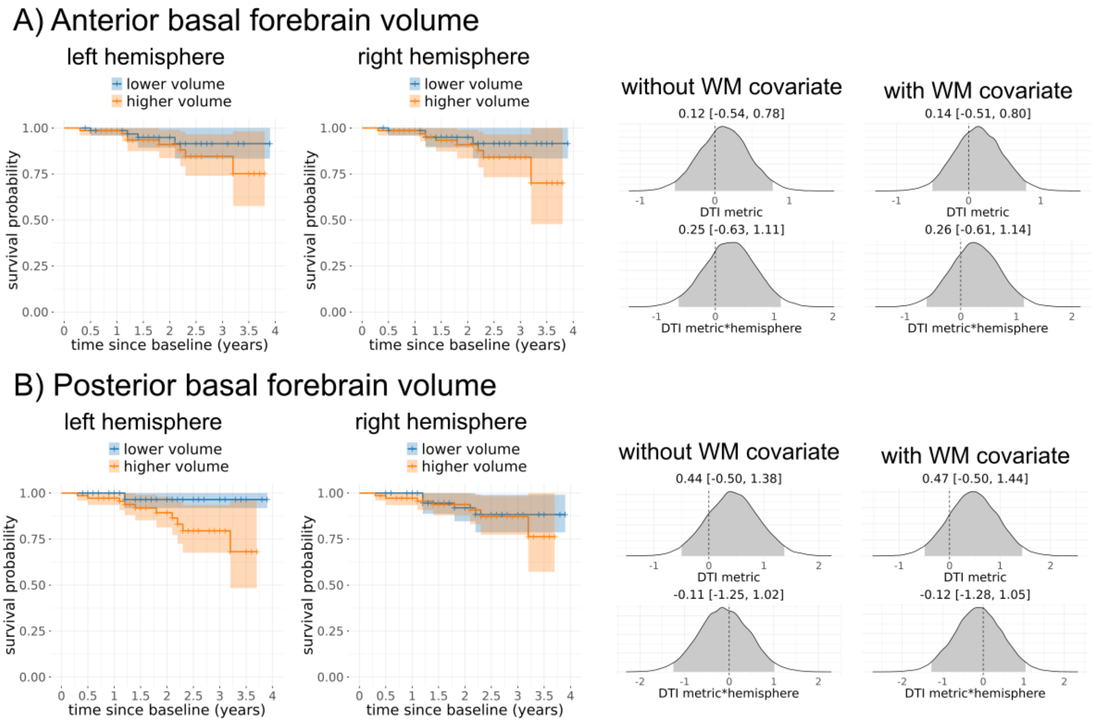

Supplementary Figure S4: Associations of basal forebrain volume with phenoconversion. Survival curves of conversion to LBD, given baseline volume, based on a median split for visualisation purposes. Also shown are posterior distributions of the standardised parameter estimates for the main effect and the interaction with hemisphere from the Bayesian Cox proportional hazards model. Shaded areas in the posterior distribution plots indicate the 95% central values of the distribution (credible interval) and above each plot the median and lower and upper borders of the 95% credible interval are stated. The zero point is indicated by a dashed vertical line.

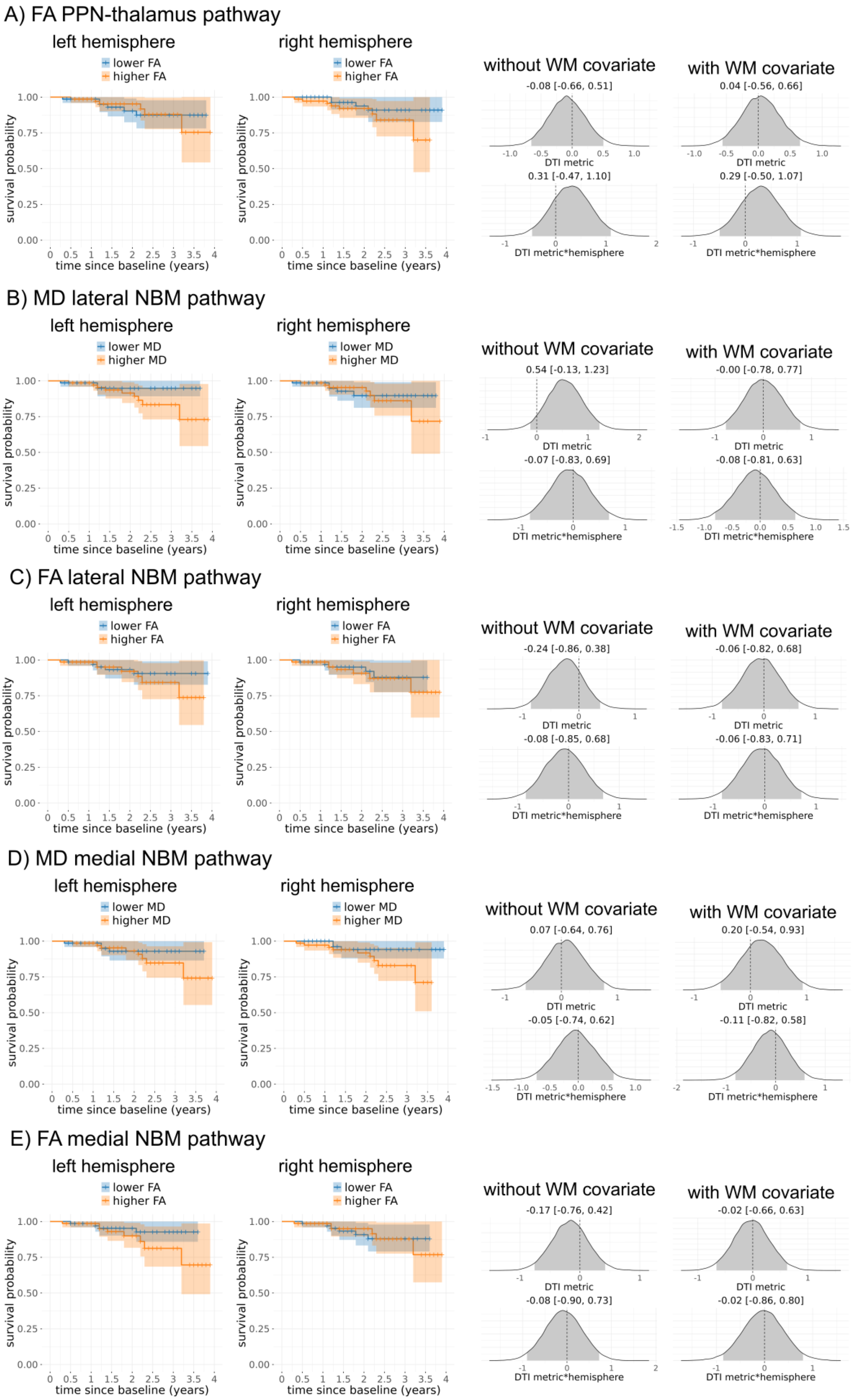

Supplementary Figure S5: Associations of pathway integrity with phenoconversion. Survival curves of conversion to LBD, given baseline MD/FA values along the respective pathways, based on a median split for

visualisation purposes. Also shown are posterior distributions of the standardised parameter estimates for the main effect and the interaction with hemisphere from the Bayesian Cox proportional hazards model. Shaded areas in the posterior distribution plots indicate the 95% central values of the distribution (credible interval) and above each plot the median and lower and upper borders of the 95% credible interval are stated. The zero point is indicated by a dashed vertical line.

FA, fractional anisotropy; MD, mean diffusivity; NBM, nucleus basalis of Meynert; PPN, pedunculopontine nucleus

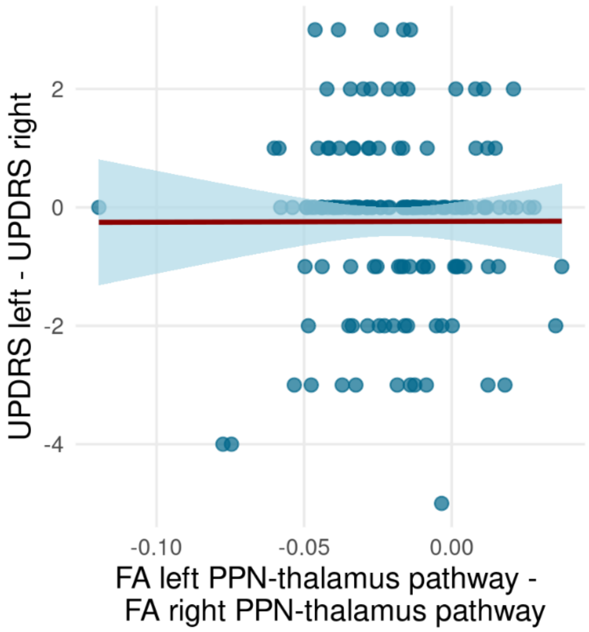

Supplementary Figure S6: Association between PPN-thalamus asymmetry and motor asymmetry in the iRBD group.
